# Comparative assessment of the efficacy of closed helical loop and T-loop for space closure in lingual orthodontics—a finite element study

**DOI:** 10.1186/s40510-018-0210-8

**Published:** 2018-05-28

**Authors:** Ajay Chacko, Tripti Tikku, Rohit Khanna, Rana Pratap Maurya, Kamna Srivastava

**Affiliations:** Department of Orthodontics and Dentofacial Orthopedics, Babu Banarasi Das College of Dental Sciences (BBDCODS), Lucknow, India

**Keywords:** Lingual orthodontics, T-loop, Closed helical loop, FEM, M/F ratio

## Abstract

**Background:**

Retraction in lingual orthodontics has biomechanical differences when compared to labial orthodontics, which is not yet established. Thus, we have intended to compare the biomechanical characteristics of closed helical loop and T-loop on 1 mm activation with 30° of compensatory curvatures during retraction in lingual orthodontics.

**Methods:**

STb lingual brackets were indirectly bonded to maxillary typhodont model that was scanned to obtain FEM model. Closed helical loop (2 × 7 mm) and T-loop (6 × 2 × 7 mm) of 0.016″ × 0.016″ TMA wire were modeled without preactivation bends. Preactivation bends at 30° were given in the software. Boundary conditions were set. The force (F) and moment (M) of both the loops were determined on 1 mm activation, using ANSYS software. M/F ratio was also calculated for both the loops.

**Results:**

T-loop exerted less force, thus increased M/F ratio as compared to closed helical loop on 1 mm activation.

**Conclusions:**

When torque has to be preserved in the anterior segment during retraction in lingual orthodontics, T-loop can be preferred over closed helical loop.

## Background

Retraction or space closure after extraction in labial as well as lingual orthodontics can be done either by friction/sliding mechanics or frictionless/loop mechanics. The drawback of sliding mechanics in terms of overcoming the amount of friction generated between the bracket and the wire interface [1], before bringing effective tooth movement, can be avoided in frictionless/loop mechanics.

Lingual orthodontics provides complete solution for patient’s esthetic concern. Lingual technique has biomechanical differences from the labial technique due to the difference in point of application of force and its distance from center of resistance (CRes) of the tooth [2]. There is tendency for retroclination of anterior teeth during retraction in lingual orthodontics as site of force application is lingual to CRes [3–5]. Hence, to overcome this, torque loss and bowing effect certain degrees of compensatory curves in addition to what is given in labial technique are incorporated in the archwire to generate counterbalancing moments [6, 7].

In contemporary labial orthodontics, many closing loops are being used for retraction such as a vertical closing loop, teardrop loops, T-loops, L-loops, mushroom loops, opus loops, keyhole loop, and open-vertical loop [8–11] whereas in lingual technique, closed helical loop, L-loop, or T-loop is commonly used for space closure [12].

To estimate the efficacy of any loop in a clinical situation, it is important to determine its biomechanical characteristics like force, moment, and moment to force ratio [13, 14]. The biomechanics of tooth movement is based on the moment of force (MF) applied on the bracket which is generated due to application of force away from the center of resistance (CRes) (MF = Force × perpendicular distance of the bracket from CRes). M/F ratio obtained as a ratio between counterbalancing moment (M_c_) given to negate the unwanted effect of MF and the force. This ratio determines the type of tooth movement possible like M/F ratio of 7:1 denotes tipping movement and 10:1 is seen in bodily and 12:1 in root movement.

The efficacy of the loops in the labial technique has been extensively researched in the last few decades [8–11]; however, there is no literature about the application of these loops in the lingual technique till date. Closed helical loop was simple in its design, and T-loop provided better torque control in anterior teeth in a clinical study cited in book by Takemoto [15]. Hence, it was decided to determine biomechanical properties of T-loop and closed helical loop in the present study.

Quantitative determination of the biomechanical characteristics of loops is not possible clinically; however, these mechanical properties can be determined by newer and precise examination tools, i.e., finite element method (FEM). FEM is a computer simulation technique used to analyze stress distribution in objects [16]. It generates a three-dimensional model with the freedom to simulate and study orthodontic force systems in all the anatomical dimensions making it possible to study statistically indeterminate system. The FEM principle is based on the division of a complex structure into smaller sections called elements in which physical properties, such as the modulus of elasticity, are applied to indicate the object response against an external stimulus such as an orthodontic force. Considering all this, the aim of this study was to evaluate and compare the force, moment, and moment to force ratio between closed helical loop and T-loop at 1 mm activation in lingual orthodontics using finite element method.

## Methods

This comparative in-vitro study was conducted by our department, in collaboration with FEA Solutions, Mumbai.

**Fig. 1:**
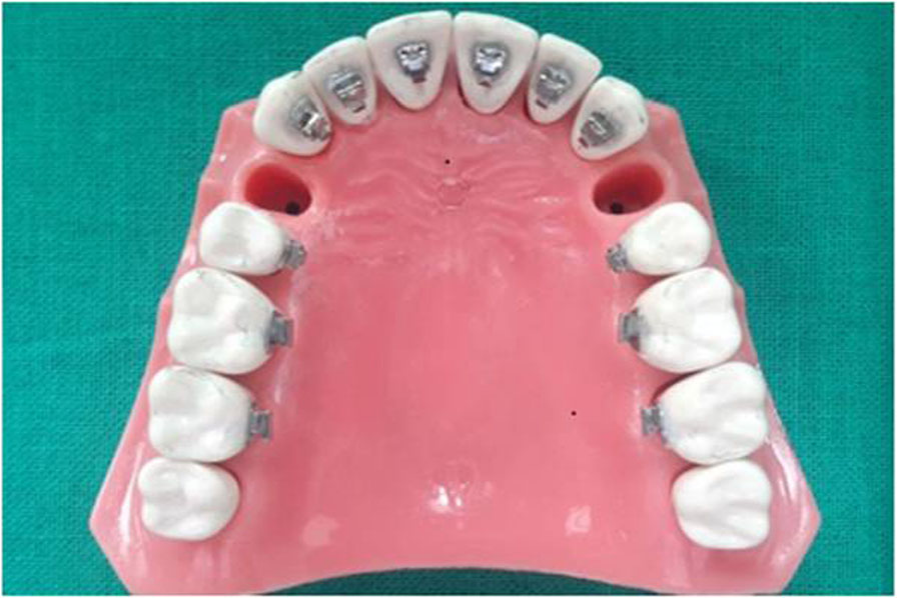
Typhodont with lingually bonded brackets and buccal tube

As this study was done only on the maxillary arch, a commercially available typhodont model of maxillary dentition in normal occlusion was selected where the 1st bicuspid was removed to simulate the extraction space needed for retraction of anterior teeth. The STb lingual brackets and molar tube with 0.18 slot, manufactured and marketed by Ormco Corporation, were selected, and transfer tray was fabricated using bracket placement device (BPD) and torque angulation device (TAD) at lab for lingual orthodontics (Fig. 1). This transfer tray was used to bond the brackets and molar tube on typhodont.

0.016″ × 0.016″ preformed titanium molybdenum alloy (TMA; ORMCO product) wires were used to fabricate closed helical loop of 2 mm width and 7 mm height and T-loop of 6 mm length, 2 mm width, and 7 mm height for this study. Both the loops were placed at the center of extraction space.

**Fig. 2:**
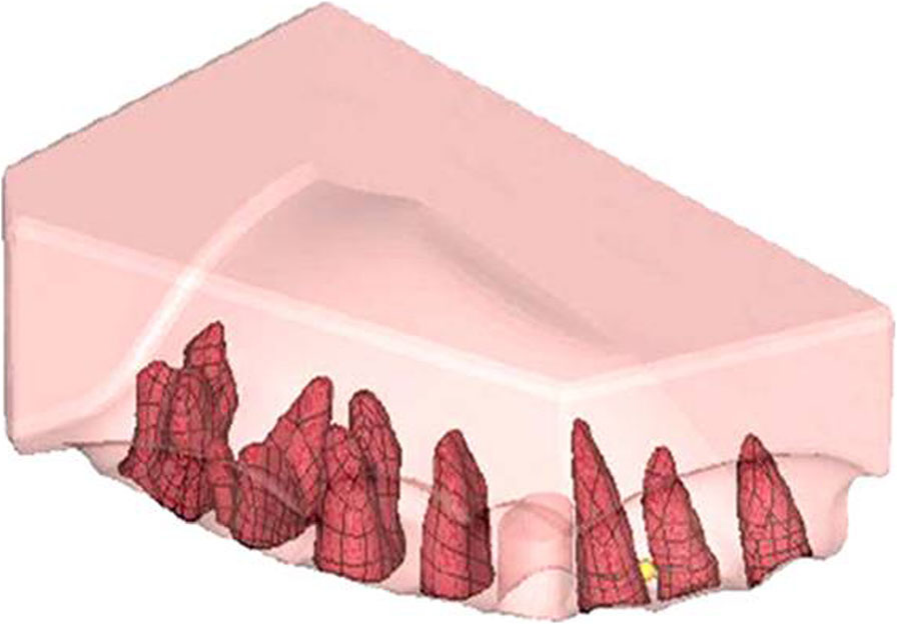
Root simulated in the software

The assembled physical typhodontic model was 3D scanned at Advance CAD Technology Pvt. Ltd., Pune, using Rexcan III 3D White light Scanner manufactured by SOLUTIONIX, Korea (2012), without preactivation bends. The CAD modeling was carried out by software named Solidwork CAD software 2014. The geometric model was constructed of the tooth with the bonded brackets in Geomagic Modelling Software, 3D Systems, Inc., USA (2014). The roots of the teeth were fabricated according to the dimensions cited in the textbook titled “Ash’ Dental Anatomy, Physiology and Occlusion” by Wheeler’s [17] (Fig. 2). Various material properties [18] prescribed for the elements of the jaw such as tooth, PDL, and alveolar bone were assigned to the geometric model to obtain the final FEM model (Table 1).

**Table 1:** Material properties of various anatomical structures

Components	Density (g/mm^3^)	Young’s modulus (GPa)	Poisson’s ratio (μ)
Teeth	1.7–06	2.03 + 04	0.3
Periodontal ligament (PDL)	1.7–06	0.667	0.49
Alveolar bone	1.7–06	1.37 + 04	0.38

**Fig. 3:**
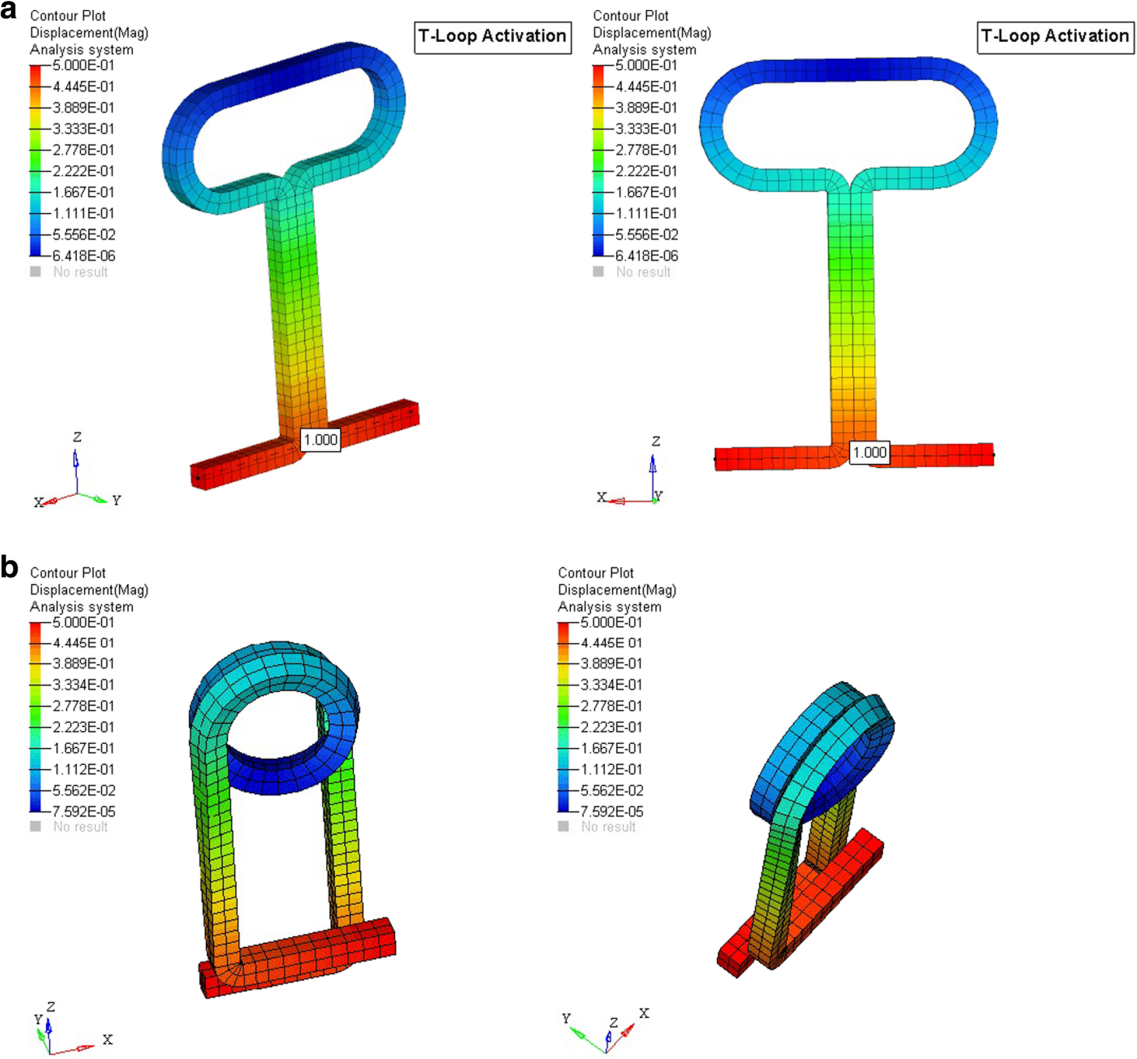
Loops simulated by 3D Hex Mesh software. **a** Closed helical loop. **b** T-loop

**Fig. 4:**
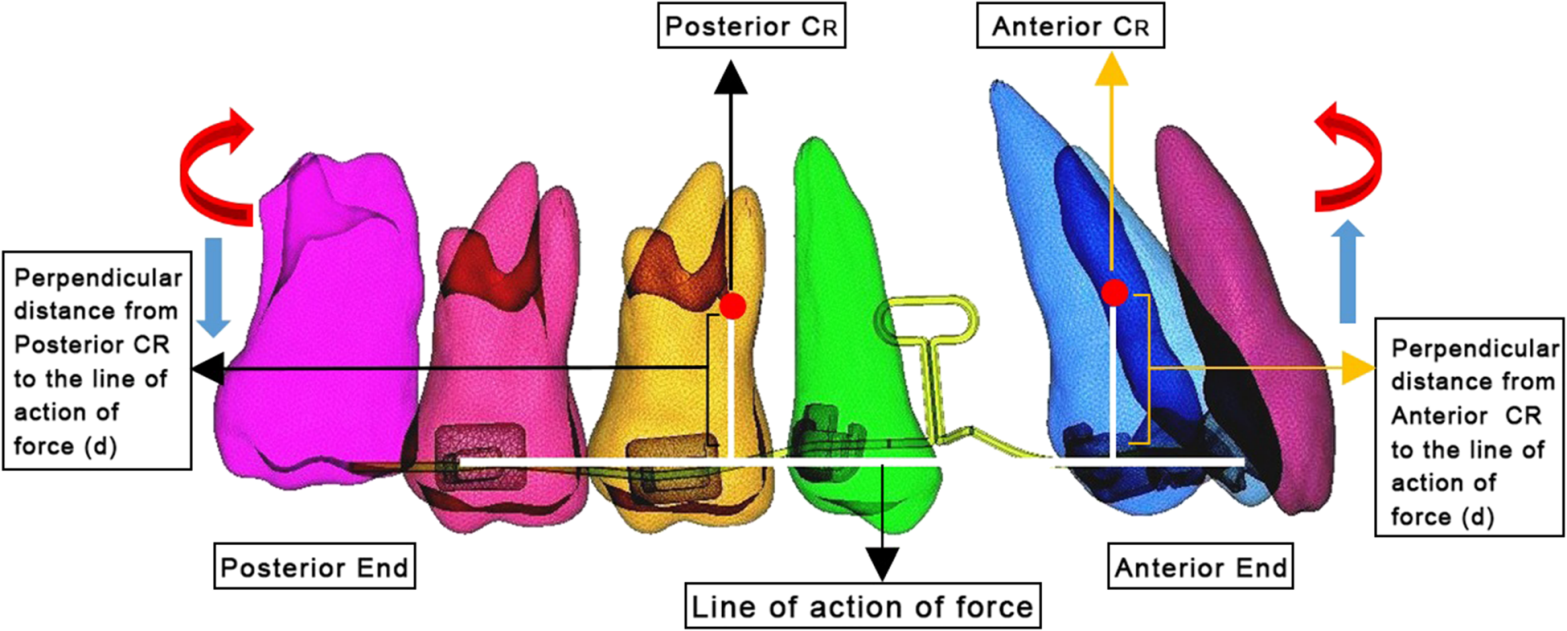
Perpendicular distance (*d*) from CRes to the line of action of force

Now, the mesh model was created by Altair hypermesh software (Altair Engineering, Inc.) after the nodal connectivity and equivalence was completed. The model of the archwire with loop was simulated separately by the 3D Hex Mesh software, CoreTech System Co., Ltd. (Figs. 3 and 4). The specific material properties [19] of stainless steel and TMA were assigned to brackets and archwire respectively (Table 2). Preactivation bends, i.e., anti-bowing curve (to prevent transverse bowing) and compensatory curves in the posterior segment, were standardized to 30°.

**Table 2:** Material properties of wire and brackets

Components	Young’s modulus (GPa)	Poisson’s ratio (μ)
TMA (ORMCO)	66	0.3
Stainless steel brackets	168	0.3

**Fig. 5:**
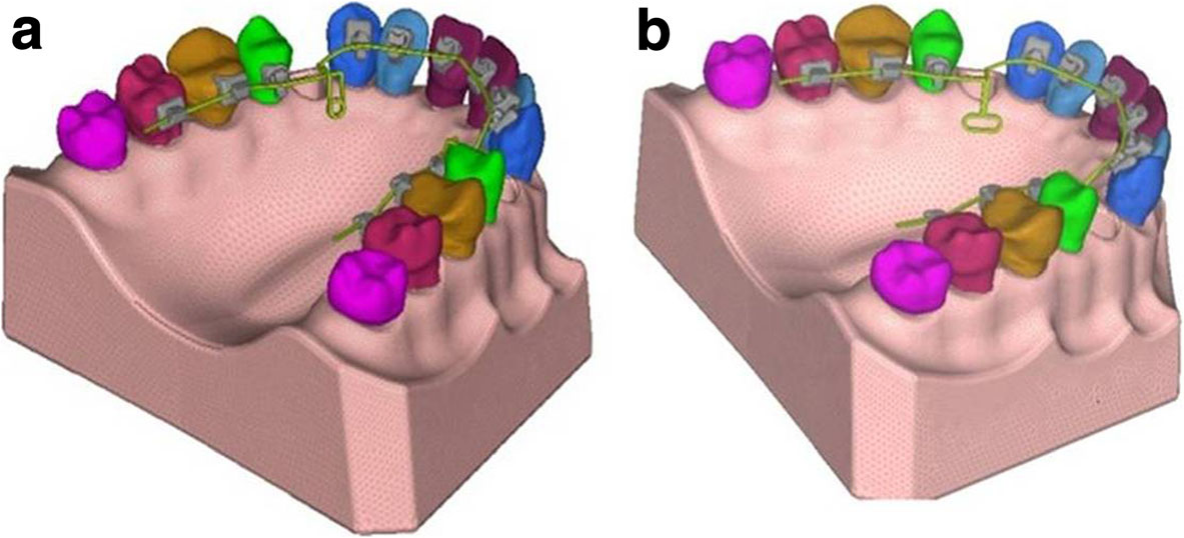
FEM model. **a** T-loop. **b** Closed helical loop

**Fig. 6:**
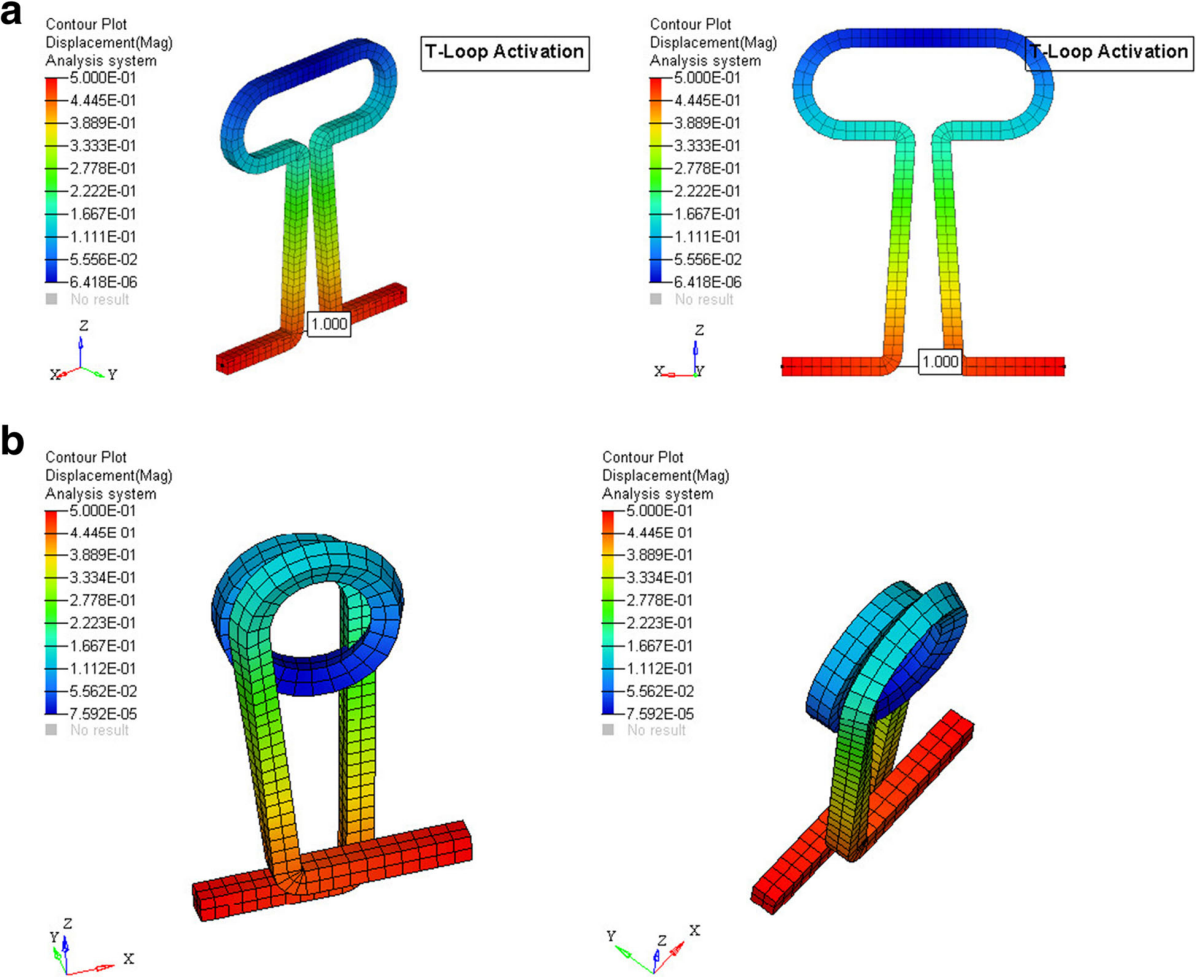
**a** Activation of T-loop. **b** Activation of closed helical loop

The center of resistance of upper 6 anterior teeth was taken 13.5 mm apical and 12.0 mm posterior from the incisal tip of the central incisors, and the center of resistance of posterior segment comprising of the 2nd premolar and 1st molar was taken mesial to the furcation of the 1st molar on its mesiobuccal root [20, 21]. This was used to determine the moment (Fig. 5). The boundary conditions were set so that the terminal node in the anterior segment was restrained in *X* and *Y* axis (i.e., it was not able to move and rotate in *X* and *Y* axis), only the rotation along the *Z* axis was allowed. The terminal node of the posterior segment was restrained in a similar way to the anterior segment, except that it was free to move along the horizontal leg of the posterior segment. This simulated the movement of the wire sliding through a molar tube. The loops were activated by the displacement of the distal end by 1 mm for both the loops, and the force and moments were obtained at both anterior and posterior end using the ANSYS software by Swanson Analysis Systems, Inc. (SASI) (Fig. 6a and b).

## Results

Nodes and elements are listed in Table 3 for this FEM study. Force reaction (F) and moment (M) was more in closed helical loop than in T-loop on 1 mm activation at 30° of compensatory curvature in posterior segment (Table 4).

**Table 3:** Nodes and elements in FEM model

Components	No. of nodes	No. of elements
Teeth	172,888	798,462
Periodontal ligament	20,429	30,211
Alveolar bone	16,684	817,296
Brackets	24,006	94,038
Archwire	6597	2928

**Table 4:** Force (*F*), moments (*M*), and *M*/*F* ratio on 1 mm activation of T-loop and closed helical loop with compensatory curvature

Loops	Nodes	*F* (g)	Moment (g mm)	*M*/*F*
T-loop	Anterior	105.6	407.62	3.86:1
	Posterior	72.29	96.87	1.34:1
Closed helical loop	Anterior	143.2	461.11	3.22:1
	Posterior	100.1	102.11	1.02:1

M/F ratio was more for T-loop than for closed helical loop (Table 4).

## Discussion

Mechanical properties of loops depend on many factors like loop design, wire materials, wire dimension, and gable bend. Earlier, the loops were made from stainless steel (SS) wires, but increased stiffness of SS wires required the use of additional length that was achieved by increasing the height of loop. This was uncomfortable for the patient at times. Hence, TMA (Beta Titanium) wires with reduced modulus of elasticity, stiffness, and load deflection rate have become the wire of choice for fabricating the loops.

The most important mechanical characteristic of a loop which determines the type of tooth movement is the moment/force ratio (M/F ratio) [12, 13]. As TMA wires exert lesser force than SS wires for the same amount of activation, hence, M/F ratio will be more in loops fabricated by TMA wires. Higher dimension TMA wires used in prescribed slot result in lesser amount of play and better torque expression that is further reinforced by gable bends given anteriorly in loops. This results in increased counterbalancing moment, hence better M/F ratios with higher dimension wires. This justifies the use of TMA wires of higher dimension in 0.18 slot over SS wires in the present study. According to the orthodontic literature, M/F ratio of 5:1 produces uncontrolled tipping, ratio of 7:1 produces controlled tipping, ratio of 10:1 produces bodily movement, and ratios greater than 10:1 produces root movement in labial orthodontics [13].

The result obtained in this study showed T-loop exerted less force, and thereby increased M/F ratio as compared to closed helical loop on 1 mm activation.

Initially, the FEM studies by Liang et al. [3] and Mascarenhas et al. [4] in 2014 concentrated on retraction of single tooth in lingual orthodontics and observed more of lingual tipping in lingual orthodontics. Similarly in a previous FEM study by Lombardo et al. [21], loss of torque control during retraction in extraction patients is more likely to occur in lingual orthodontics than in labial using sliding mechanics.

Several studies had been conducted to assess biomechanical properties of the loops, used for anterior retraction in labial orthodontics, but no such attempt had been done so far in lingual orthodontics. In 2006, Safavi et al. [8] conducted a study to compare biomechanical characteristics of T-loop, vertical helical loop, L-loop, and opus loop of 0.016 × 0.022 wire of stainless steel but did not consider the modeling of brackets (whether lingual or labial) or tooth along with the root or the compensatory curvatures. They obtained higher force in their study as they did not consider placement of wire in the brackets. Hence, the moment obtained by them was also higher and was not truly representative of moment obtained during orthodontics tooth movement. The M/F ratio of T-loop (13.4) was also higher in their study because of the difference in the loop design and length, difference in the material used for fabricating the loop (made of stainless steel), degree of compensatory curvatures, and importantly the fact that their study was on labial orthodontics. Such higher M/F ratios at anterior end in their study are representative of root movement that is difficult to attain on 1 mm activation in reality.

Yet another study has been conducted by Patel et al. [10] in labial orthodontics comparing the biomechanical properties of T-loops, mushroom loops, teardrop loop, and keyhole loop of 0.019 × 0.025 TMA on 2 mm activation at 4 tooth nodes (incisors, canine, premolar, and molar tooth node). They used the tooth and the bracket to determine the interbracket distance; later, they excluded both tooth and brackets and just considered the wire for study. M/F ratio showed variable values. This could probably be due to difference in the range of activation, measuring the force at tooth nodes instead of anterior and posterior end of loop as used in present study.

Amongst the various variations in position, amount of deflection, height, length, and width of T-loop in a study by Chaudary et al. [22] in 2013, the biomechanical properties of T-loop of height 7 mm made of 0.017 × 0.025 TMA placed in the center of extraction space showed variable results in terms of force and M/F ratio.

Techalertpaisarn et al. [23] conducted a study in 2013, assessing the mechanical properties of opus closing loops, L-loops, and T-loops at a distance of 2, 4, 6, 8, and 10 mm from premolar brackets with a interbracket distance of 12 mm and on application of 100 and 200 g of horizontal force. The authors stressed on the importance of the shape of loop to adjust its mechanical properties. Loop height affects M/F ratio, i.e., as loop height increased, M/F ratio increased, but no loop can attain M/F ratio greater than its height. Even Burstone and Koenig reported that height matters more than length of the loop [24]. In this present study, the M/F ratio of T-loop in both the conditions is lesser than the loop height, i.e. 7 mm in the present study, and the same was true for closed helical loop.

Techalertpeisarn et al. [25] also conducted another FEM study in 2016 to compare the mechanical properties of T-loop force system with and without vertical step fabricated on 0.016 × 0.022 stainless steel wire. They have used 0.018 slot bracket and measured the M/F ratio at canine and premolar brackets. They observed the M/F ratio increased on increasing loop height and length from 8 to 10 mm, increasing the inter bracket distance from 6, 9, to 12, increasing the vertical step, and decreasing the force of activation.

Various authors had used different techniques to determine the biomechanical properties of loop during tooth retraction, besides FEM [6, 26–28]. In 2016, Srivastava et al. [26] used Loop software program (dHal) to calculate force and moment and their ratios at various positions and for various activations for a standard design of T-loop and found comparable results.

In another study by Kum et al. [29], M/F ratio of 3 closing loops U-, T-, and X-loop was measured during activation and deactivation using force and moment transducers in labial orthodontics. They found lesser values of M/F ratio, as they did not incorporate any gable bend in the legs of the loop.

Although FEM methods allow the evaluation of detailed behavior of different types of loops in terms of force, moment, displacements, and stress by simulating a clinical condition of tying loops to the brackets and activating it, this approach has its own limitations. FEM does not allow us to study the changes in the force system or the stress pattern as the wire deactivates or as the tooth moves under the influence of the forces. When teeth or groups of teeth move to new positions during orthodontic treatment, interbracket distance, bracket angulation, vertical position, and loop activation will change gradually. These changes will alter the loop conditions and thus potentially the mechanical properties. Even the linear properties of PDL are taken to be isotropic in FEM studies whereas the histological changes in PDL on application of orthodontic force can alter its material properties [10, 22, 30].

Despite of these limitations of FEM analysis, the result of this study indicates that T-loop showed more M/F ratio than closed helical loop at 30° of compensatory curvature (Table 4). These results can be applied in different clinical situations when using lingual technique where chances of lingual tipping are always more in comparison to labial technique. When severely proclined incisors have to be retracted in lingual orthodontics, then T-loop or closed helical loop can be used, and as the teeth uprights, there will be gradual decay of force, thereby increasing the M/F ratio at anterior end. When torque has to be preserved from beginning in anterior segment during retraction, T-loop with better M/F ratios can be preferred over closed helical loop. In future, FEM studies can be conducted to assess the mechanical properties of different loops in different lingual bracket systems or the effect of loop shape, size, and position on retraction in lingual orthodontics can be done. As there is no published data for the numerical values of M/F ratio for various tooth movements in lingual orthodontics, the same can be determined in the future.

The horizon of further studies can be expanded to include the assessment of mechanical properties of loop under changing condition as the teeth moves to newer position during retraction and results of FEM approach must be correlated with clinical experiments to validate its findings.

## Conclusions

1. Closed helical loop delivered more force and moment of force as compared to T-loop at both anterior and posterior ends of the loop on 1 mm activation with 30° of compensatory curvature.
2. The M/F ratio was found to be higher in T-loop than in closed helical loop at 30° of compensatory curvature.
